# Injuries Reported by Selected Health Facilities During the Arbaeenia Mass Gathering at Babel Governorate, Iraq, 2014: Retrospective Records Analysis

**DOI:** 10.2196/10877

**Published:** 2020-05-28

**Authors:** Abdulaal Chitheer, Faris Lami, Ahmed Radhi, Ali Arbaji

**Affiliations:** 1 Misan Directorate of Health Iraq Ministry of Health Misan Iraq; 2 Department of Community and Family Medicine College of Medicine University of Baghdad Baghdad Iraq; 3 Injury Surveillance Program Iraq Ministry of Health Baghdad Iraq; 4 Eastern Mediterranean Public Health Network (EMPHNET) Amman Jordan

**Keywords:** mass gathering, Injury, Karbala, Iraq

## Abstract

**Background:**

Arbaeenia is the largest religious mass gathering in Iraq. The conditions associated with mass gatherings result in high rates of injury. There have been no prior studies on injuries during the Arbaeenia mass gathering.

**Objective:**

This study describes the injuries observed during the Arbaeenia mass gathering in Babel Governorate in Iraq between November 24 and December 14, 2014.

**Methods:**

The study was conducted in Babel Governorate at the emergency departments of six public hospitals and two major temporary medical units that were located along the three roads connecting the Middle and Southern Iraqi governorates. We used the Iraq Injury Surveillance System modified form to collect information on injured patients treated in the selected facilities. Data on fatal injuries was obtained from the coroner’s office. The following data were collected from the patients: demographics, outcome of injury, place and time of occurrence, mode of evacuation and medical care before arriving at the hospital, duration of travel from place of occurrence to hospital, disposition of non-fatal injury, cause and mode of injury, and whether the injury occurred in connection with the Arbaeenia mass gathering.

**Results:**

Information was collected on 1564 injury cases, of which 73 were fatal. About half of the reported nonfatal injuries, 687/1404 (48.9%), and a quarter of fatalities, 18/73 (25%) were related to the Arbaeenia mass gathering (*P*<.001). Most of the reported injuries were unintentional, 1341/1404 (95.51%), occurred on the street, 864/1323 (65.6%), occurred during the daytime 1103/1174 (93.95 %). Most of those injured were evacuated by means other than ambulance 1107/1206 (91.79%) and did not receive pre-hospital medical care 788/1163 (67.7%). Minor injuries 400/1546 (25.9%) and traffic accidents 394/1546 (25.5%) were the most common types of injuries, followed by falls 270/1546 (17.5%). Among fatal injuries, traffic accidents 38/73 (52%) and violence 18/73 (25%) were the leading causes of death. Mass gathering injuries were more likely to occur among individuals aged 21-40 years (odds ratio [OR] 3.5; 95% CI 2.7-4.5) and >41 years (OR 7.6; 95% CI 5.4-10.6) versus those <21 years; more likely to be unintentional than assault (OR 5.3; 95% CI 1.8-15.5); more likely to happen on the street versus at home (OR 37.7; 95% CI 22.4-63.6); less likely to happen at night than during the day (OR 0.2; 95% CI 0.1-0.4); and less likely to result in hospital admission (OR 0.5; 95% CI 0.3-0.7).

**Conclusions:**

The study shows that most injuries were minor, unintentional, and nonfatal, and most people with injuries had limited access to ambulance transportation and did not require hospitalization.

## Introduction

Mass gatherings are defined as events with an attendance of more than 1000 persons or as events attended by more than 25,000 people [1]. Injuries that occur during mass gatherings are often due to overcrowding, stampedes, terrorism, and spiritual acts [2,3]. Injuries and noncommunicable diseases are responsible for more deaths and morbidity during mass gatherings than communicable diseases [4].

During the annual Hajj, trauma is a major cause of injury and death. Pilgrims walk long distances through or near dense traffic and motor vehicle accidents are inevitable; however, the most feared trauma hazard is a stampede [5]. In Iraq, injuries are the second leading cause of death and violence, while road traffic, fire, and drowning are among the main causes of injury-related deaths [6,7]. In 2010, the Iraq Injury Surveillance System (IISS) was established in major hospitals and coroners’ offices to enable timely electronic reporting of injuries. Al-Hilla hospital, a major referral hospital in Babel Governorate, and the Babel Coroner’s Office were added to the roll of IISS reporting facilities in 2013 [8].

The ability of the Iraq health system to respond to injuries is challenged during mass gatherings, during which the risk of injury increases. The Arbaeenia mass gathering is the largest gathering of Shia Muslims worldwide and occurs annually in Karbala, Iraq. During this mass gathering, approximately 20 million pilgrims from nearly 40 countries attend the ceremony [9,10]. Many of the pilgrims travelling to Karbala walk a distance of up to 600 kilometers through several Iraqi governorates, and millions of Arbaeenia attendees pass through Babel Governorate along the three roads connecting Middle and Southern Iraqi governorates to Babel.

There are few studies on the public health problems associated with mass gatherings [11]. During a mass gathering, road traffic injuries and terrorism are major risks to the health of pilgrims and the local community [12]. Because of cultural or religious beliefs, some attendees practice self-harm such as laceration of their scalp using sharp knives and other risky practices [11]. There is particularly limited information available on injury surveillance systems at mass gatherings [13]. The importance of developing public health surveillance system in mass gatherings has been emphasized in recommendations from previously published reports [12,14]. Public health for mass gatherings health is an evolving niche of prehospital care rooted in emergency medicine, emergency management, public health, and disaster medicine [15].

This study describes the mass gathering injuries reported at selected health facilities in Babel Governorate in Iraq during the Arbaeenia mass gathering in 2014.

## Methods

We conducted this study in Babel Governorate between November 24, 2014 and December 14, 2014. The emergency departments (ED) of six public hospitals in six districts and two major temporary medical units were selected for convenient data collection. The selected facilities were located along the three roads connecting the Middle and Southern Iraqi governorates to Babel Governorate.

We used an IISS modified form to collect information on injury cases treated in the selected facilities. Data on fatal injuries were obtained from the coroner’s Office in IISS sentinel sites in the selected areas. The following data were collected: patient demographics, injury outcome, place and time of occurrence, mode of evacuation and medical care before arriving the hospital, duration of travel from place of occurrence to hospital, disposition of nonfatal injury, cause and mode of injury, and association with the Arbaeenia mass gathering.

Data entry was performed using Epi Info and SPSS Statistics was used for data analysis. We estimated the injury frequencies and percentages by demographics and odds ratios of the factors associated with injuries at the mass gathering. Chi-square statistics were used to test significance at *P*<.05. We estimated the 3-period moving average for the daily trend of fatal and nonfatal injuries to remove trend fluctuations.

## Results

There were 1564 injuries treated in the health facilities selected for this study. Of these, 687/1404 (48.9%) were injuries related to the Arbaeenia mass gathering; of the 73 (5%) fatal injuries, 18/73 (25%) were related to the mass gathering. The majority of the injuries, 1096/1564 (70.1%), were collected from the ED of six public hospitals, 395/1564 (25.2%) were from the two temporary health facilities, and 73/1564 (5%) were from the coroners’ offices. A major proportion of victims were aged less than 21 years old (44.8%, 685/1564), 72.6% (1136/1564) were males, and 63.4% (955/1564) were residents of Babel Governorate (Table 1).

**Table 1:** Demographics of patients with sustained injuries during the Arbaeenia mass gathering in Babel Governorate, Iraq, 2014 (N=1564)^a^.

Characteristics		n (%)
**Reporting sites**		
	Hospitals (emergency departments)	1096 (70.1)
	Temporary health care facilities	395 (25.3)
	Coroner’s office	73 (4.7)
	Total	1564
**Injury related to mass gathering**		
	Yes	687 (48.9)
	No	717 (51.1)
	Total	1404
**Age groups (years)**		
	<21	685 (43.8)
	21-40	571 (36.5)
	≥41	279 (17.9)
	Total	1535
**Sex**		
	Male	1136 (72.6)
	Female	428 (27.4)
	Total	1564
**Place of residence**		
	Babel Governorate	955 (63.4)
	Other Iraqi Governorates	534 (35.4)
	Other countries (Iran, Saudi Arabia, Turkey, Afghanistan)	17 (1.2)
	Total	1506

^a^ Totals may be <1564 due to missing data.

Table 2 shows the location and time of injuries, factors related to medical services, and relationship to the mass gathering. Most injuries (864/1564, 55.2%) occurred on the street and 580/680 (67.2%) of these were mass gathering-related. Approximately 93.9% (1103/1174) of injuries occurred during the day, and 605/619 (54.9%) of these associated with the mass gathering. The injuries were predominantly unintentional 1341/1404 (95.5%). Only 17/1404 (1%) of injuries were intentional (self-inflicted) and 16/687 (94%) of these were related to the mass gathering. Ambulance services were used for only 99/1206 (8%) of injuries, whereas 661/1206 (54.8%) and 446/1206 (36.9%) were transported in other vehicles or carried directly by people to the hospital. The majority 749/1186 (63.2%) of the injured people reached the hospital within an hour of injury, 258/1186 (21.7%) reached between 2 hours and 24 hours, and 179/1186 (15.1%) reached after >24 hours. Of the injuries that reached the hospital after more than an hour, 208/623 (80.6%) – 168/623 (93.9%) were injuries related to the mass gathering. Only 375/1163 (32.3%) of injuries were medically treated before reaching the hospital, 333/603 (88.8%) of these associated with the mass gathering; and 148/1133 (11%) of injuries were admitted to the hospital, 55/667 (38%) of these were related to the mass gathering.

**Figure 1:**
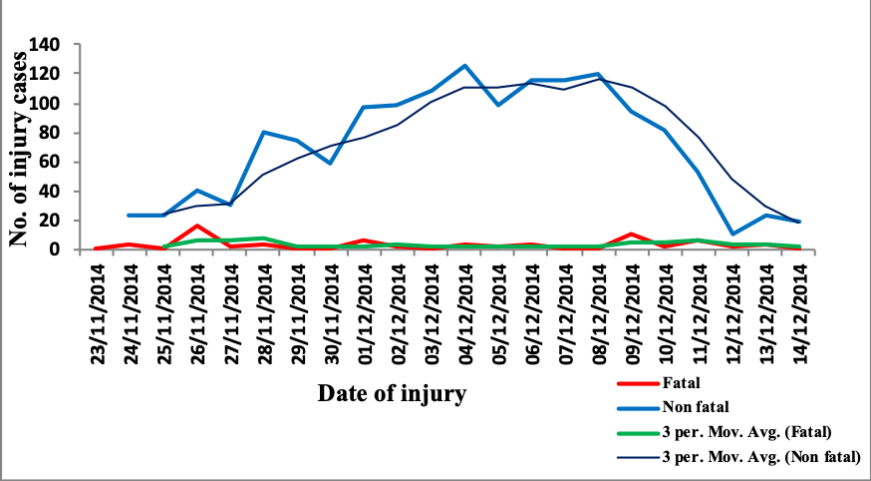
Trend of reported injuries during Arbaeenia mass gathering in Babel Governorate, Iraq, 2014 (N=1561).

The moving average daily trend for nonfatal injuries showed a gradual increase from the start of the study on November 24 and peaked on December 8, then declined prior to the day of the Arbaeenia celebration (December 13, 2014). The daily trend for fatal injuries was constant throughout the period, using the moving average (Figure 1).

**Figure 2:**
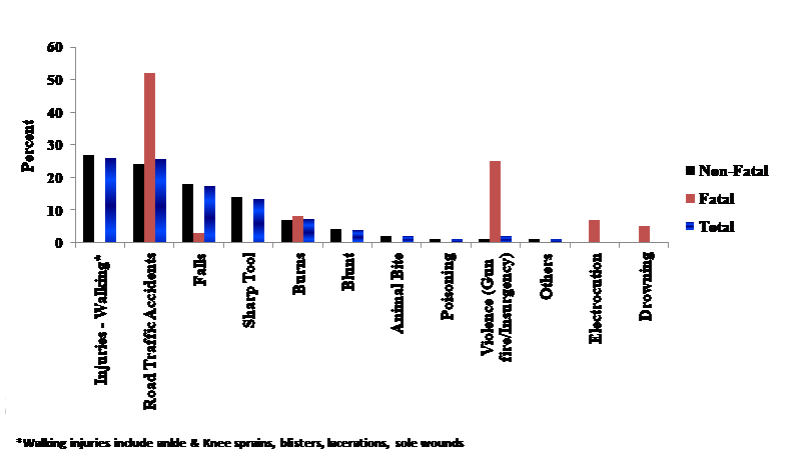
Distribution of causes of injuries by outcome during the Arbaeenia mass gathering in Babel Governorate, Iraq, 2014 (N=1564).

Figure 2 shows the distribution of injury causes. Of the fatal injuries, 52% were due to traffic accidents and 25% were due to gun violence. Of the nonfatal accidents, the leading causes were injuries related to walking (27%), traffic accidents (24%), and falls (18%).

Table 3 shows the factors associated with injuries incurred during the Arbaeenia mass gathering injuries. Fatal injuries were less likely to be associated with the mass gathering (OR 0.3; 95% CI 0.2-0.4) compared to nonfatal injuries. Compared to the people in the <21 years age group, those 21-40 years of age (OR 3.5; 95% CI 2.7-4.8) and >40 years of age (OR 7.6; 95% CI 5.4-10.6) were more likely to be injured in the mass gathering. Injuries among women were more likely to be associated with the mass gathering (OR 1.4; 95% CI 1.1-1.8). Compared to injuries from assault, self-inflicted and unintentional injuries were more likely to be associated with the mass gathering (self-inflicted: OR 88, 95% CI 9-863; unintentional: OR 5.3, 95% CI 1.8-15.5). Compared to injuries that occurred at home, injuries that occurred in the street, at work, and elsewhere were more likely to be associated with the mass gathering (OR 37.7, 95% CI 22.4-63.6; OR 25.7, 95% CI 14.1-47.3; and OR 9.2, 95% CI 2.5-33.8, respectively). Injuries that occurred at night were less likely to be mass gathering-related than those that occurred during the day (OR 0.2; 95% CI 0.1-0.4). MG injuries were more likely to be evacuated by other means (e.g., carried by humans; OR 19.8, 95% CI 11-35.8) than by ambulance. People with injuries who took 2-24 hours and >24 hours to reach a hospital were more likely to have mass-gathering-related injuries (OR 8.5; 95% CI 6-11.9 versus OR 31; 95% CI 16.6-58.2), compared to those with injuries who reached a facility in <2 hours. Injuries that received medical care before reaching the hospital were more likely to be mass gathering-related than those that did not receive medical care (OR 15.2, 95% CI 11.1-20). Injuries admitted to the hospital were less likely to be mass gathering-related than those not admitted (OR 0.5, 95% CI 0.3-0.7).

**Table 2:** Injury characteristics and relationship to the Arbaeenia mass gathering in Babel Governorate, Iraq, 2014 (N=1564).

Variables		Relationship to the mass gathering		
		Yes (%)	No (%)	Total^a^ n (%)
**Place of occurrence**				
	Home	16 (5.2)	294 (94.8)	310 (23.4)
	Street	580 (67.2)	864 (32.8)	864 (55.2)
	Workplace	80 (58.4)	57 (41.6)	137 (10.3)
	Others	4 (33.3)	8 (66.7)	12 (1.1)
	Total	680 (51.4)	643 (48.6)	1323 (100)
**Time of occurrence**				
	Day time (6 am to 5 pm)	605 (54.9)	498 (45.1)	1103 (93.9)
	Nighttime (6 pm to 5 am)	14 (19.7)	57 (80.3)	71 (6.1)
	Total	619 (52.8)	555 (47.2)	1174 (100)
**Cause** **of** **injury**				
	Assault	4 (15.4)	22 (84.6)	26 (1.8)
	Intentional	16 (94.1)	1 (5.9)	17 (1.2)
	Unintentional	658 (49.1)	683 (50.9)	1341 (95.5)
	Unknown	9 (45.0)	11 (55.0)	20 (1.5)
	Total	687 (48.9)	717 (51.1)	1404 (100)
**Mode of evacuation**				
	Ambulance	50 (50.5)	49 (49.5)	99 (8.3)
	Other vehicles	171 (25.9)	484 (74.1)	661 (54.8)
	Other means (carried by people)	425 (95.3)	21 (4.7)	446 (36.9)
	Total	646 (53.6)	560 (46.4)	1206 (100)
**Prehospital time interval (hours)**				
	1	247 (33.0)	502 (67.0)	749 (63.2)
	2-24	208 (80.6)	50 (19.4)	258 (21.7)
	>24	168 (93.9)	11 (6.1)	179 (15.1)
	Total	623 (52.5)	563 (47.5)	1186 (100)
**Prehospital medical care**				
	Not received	270 (34.3)	518 (65.7)	788 (67.7)
	Received	333 (88.8)	42 (11.2)	375 (32.3)
	Total	603 (51.8)	560 (48.2)	1163 (100)
**Disposition of nonfatal injuries**				
	Not admitted (treated and discharged)	585 (55.7)	468 (44.3)	1053 (79)
	Admitted ^b^	55 (37.8)	93 (62.2)	148 (11)
	Unknown	27 (20.8)	103 (79.2)	130 (10)
	Total	667 (50.2)	664 (49.8)	1331

^a^Totals differ due to missing data or lack of response.

^b^Including self-discharged and referred.

**Table 3:** Factors associated with injuries occurring during the Arbaeenia mass gathering in Babel Governorate, Iraq, 2014 (N=1564)

Variables			Mass gathering-related				Total, n (%) ^a^		Odds ratio (95% CI)		*P* value	
				Yes (%)		No (%)						
**Outcome**												
	Nonfatal		670 (50.3)		661 (49.7)		1331 (94.8)		1			
	Fatal		18 (25)		55 (75)		73 (5.2)		0.3 (0.2-0.6)		<.001	
	Total		688 (49)		716 (51)		1404 (100)					
**Age (years)**												
	<21		174 (29.3)		429 (70.7)		594 (43.1)		1			
	21-40		306 (59.1)		212 (40.9)		518 (37.6)		3.5 (2.7-4.5)		<.001	
	≥41		201 (75.8)		64 (24.2)		265 (19.3)		7.6 (5.4-10.6)		<.001	
	Total		681 (49.5)		696 (50.5)		1377 (100)					
**Sex**												
	Males		474 (46.6)		544 (53.4)		1018 (72.5)		1			
	Females		213 (55.2)		173 (44.8)		386 (27.5)		1.4 (1.1-1.8)		.004	
	Total		687 (48.9)		717 (51.1)		1404 (100)					
**Intent of injury**												
	Assault		4 (15.4)		22 (84.6)		26 (1.8)		1			
	Self-harm		16 (94.1)		1 (5.9)		17 (1.1)		88 (9-863		<.001	
	Unintentional		659 (49.1)		682 (50.9)		1341 (95.6)		5.3 (1.8-15.5)		.02	
	Unknown		9 (45.0)		11 (55.0)		20 (1.5)		4.5 (1.1-17.9)		.033	
	Total		688 (49.0)		716 (51.0)		1404 (100)					
**Place of occurrence**												
	Home		16 (5.2)		294 (94.8)		310 (23.4)		1			
	Street		581 (67.2)		283 (32.8)		864 (65.3)		37.7 (22.4-63.6)		<.001	
	Workplace		80 (58.4)		57 (41.6)		137 (10.3)		25.7 (14.1-47.3)		<.001	
	Others		4 (33.3)		16 (66.7)		12 (1.0)		9.2 (2.5-33.8)		.001	
	Total		681 (51.5)		642 (48.5)		1323 (100)					
**Time of occurrence**												
	Daytime (6 am to 5 pm)		605 (54.9)		498 (45.1)		1103 (93.9)		1			
	Nighttime (6 pm to 5 am)		14 (19.7)		57 (80.3)		71 (6.1)		0.2 (0.1-0.4)		<.001	
	Total		619 (52.8)		555 (47.2)		1174 (100)					
**Mode of evacuation**												
	Ambulance		50 (50.5)		49 (49.5)		99 (8.2)		1			
	Other vehicles		171 (25.9)		490 (74.1)		661 (54.8)		0.34 (0.2-0.5)		<.001	
	Other means (carried by other people)		425 (95.3)		21 (4.7)		446 (37.0)		19.8 (11-35.8)		<.001	
	Total		646 (53.6)		560 (46.4)		1206 (100)					
**Prehospital time interval (hours)**												
	1		247 (32.9)		502 (67.1)		749 (63.1)		1			
	2-24		208 (80.6)		50 (19.4)		258 (21.8)		8.5 (6-11.9)		<.001	
	> 24		168 (93.9)		11 (6.1)		179 (15.1)		31 (16.6-58.2)		<.001	
	Total		623 (52.5)		563 (47.5)		1186 (100)					
**Prehospital medical care**												
	Not received		270 (34.3)		518 (65.7)		788 (67.7)		1			
	Received		333 (88.8)		42 (11.2)		375 (32.3)		15.2 (11.1-20)			
	Total		603 (51.8)		583 (48.2)		1163 (100)				<.001	
**Disposition of nonfatal injuries**												
	Not admitted (treated and discharged)		586 (55.7)		467 (44.3)		1053 (79.1)		1			
	Admitted		56 (37.8)		92 (62.2)		148 (11.1)		0.5 (0.3-0.7)		<.001	
	Unknown		27 (20.8)		103 (79.2)		130 (9.8)		0.2 (0.2-0.3)		<.001	
	Total		669 (48.9)		464 (51.1)		1331 (100)					

^a^Totals are different due to missing data or lack of response.

## Discussion

The study describes injuries reported at several health facilities during the Arbaeenia mass gathering in Babel Governorate, Iraq. Most of the injuries were minor (walking-related), unintentional, or nonfatal with limited or no access to ambulance transportation and the affected people did not require hospitalization. The injured individuals often reached a hospital within an hour of injury. People with injuries who took more than 1 hour to reach a hospital but received medical care prior to arrival were more common among people with injuries associated with the mass gathering than those that were not.

During mass gatherings, trauma is one of the most common medical problems [16]. The health consequences of mass gatherings include injuries resulting from crowd density and inadequate infrastructure, exposure to extreme weather events, and escalation of violence as a result of crowd behavior [17]. The injured patients during the Arbaeenia mass gathering were mostly young, consistent with the global figures on injuries [18]. Youth are more likely to take risks than older individuals, increasing their risk of injury [19]. Unintentional injuries accounted for the vast majority of cases reported, which was consistent with the findings of global and national injury surveillance reports [8].

In contrast to reports from IISS data and other sources, this study found that nearly two-thirds of the injuries occurred in the street, as opposed to in the home [8,20,21]. The high occurrence of injuries in the street is a result of the nature of the mass gathering, during which pilgrims travel long distances on foot to attend the event in Karbala.

Few of the injuries were transported to the hospital by ambulance, which could explain the lack of prehospital medical care for the majority of injuries. Despite the lack of ambulance services to evacuate the injuries, the majority of patients reached a health care facility within an hour of injury, which trauma experts consider the critical timeframe for lifesaving efforts. In general, few injury victims receive prehospital medical care and ambulance transportation [22,23]. This study showed that people with injuries related to the mass gathering were less likely to reach the hospital within the critical 1-hour timeframe. Road congestion during the mass gathering may have delayed patients in reaching the hospitals. The majority of injured patients did not require hospitalization, consistent with other religious mass gatherings and injury surveillance reports [8,11,23,24].

The ratio of injury deaths to hospital admissions and ED attendants in this study was 1:2.7:15.7, whereas the IISS report cited a ratio 1:1.5 6 [8]. Our study included minor injuries, which comprised the majority of mass gathering-related injuries, possibly increasing the observed ER burden. Traffic accidents, which accounted for half of injury deaths and a quarter of nonfatal injuries, may also have contributed to the high admission ratio. Hospital admission rates depend on the severity of injury, access to hospital services, and health system structure [25].

The majority of injuries occurred during the daytime, which is consistent with information observed in the IISS report and other studies [8,19,26]. The high occurrence of injuries during the daytime may be explained by the high traffic volume during the day. The pilgrims walk during the day and often rest in the evening, which reduces their risk of traffic accidents during the night.

Injuries among women were more likely to be mass gathering-related than those among men. Burns are common among females in Iraq and worldwide, as women have higher exposure to heat or hot surfaces during cooking, and probably this is the case in mass gathering. In contrast, men are mostly the victims of the traffic accident injuries [8,24,27].

Traffic accidents are the leading cause of fatal injuries in Iraq and globally [6]. Traffic accidents accounted for more than half of the fatal injuries in this study, which is higher than the global figures on fatal injuries (24%) [28]. This may be due to the large number of pilgrims traveling on foot and the high traffic volume on the roadways. A review of studies in low-to-middle-income countries revealed that traffic accidents accounted for one-third to four-fifths of traumatic injury admissions, one-tenth to one-third of all injuries treated in hospitals, and almost half of all bed occupancies in surgical wards [26]. This study showed similar findings on injuries treated in hospitals.

Most of the injuries reported were minor (75%), which is consistent with prior reports on injuries related to other mass gatherings and may explain why the majority of the injury cases were not admitted to the hospitals [29]. This information is consistent with a study among Iranian pilgrims during the Hajj mass gathering [30-32].

This study had several limitations. Some potential subjects may have been missed during data collection. In addition, data for some items, particularly the disposition variable, were missing. Injury data were not collected from all health facilities, which limits generalizability. Injury data collected from health facilities may have underestimated the total injuries that occurred because individuals with minor injuries may not seek medical care from the health facilities [33]. Injury rates could not be calculated, because the population at risk is unknown and could not be determined.

## Abbreviations

LMIC: low and middle-income countries
